# A spatially explicit risk assessment approach: Cetaceans and marine traffic in the Pelagos Sanctuary (Mediterranean Sea)

**DOI:** 10.1371/journal.pone.0179686

**Published:** 2017-06-23

**Authors:** Maria Grazia Pennino, Antonella Arcangeli, Vinícius Prado Fonseca, Ilaria Campana, Graham J. Pierce, Andrea Rotta, Jose Maria Bellido

**Affiliations:** 1 Statistical Modeling Ecology Group (SMEG), Departament d'Estadística i Investigació Operativa, Universitat de València, Burjassot, Valencia, Spain; 2 Instituto Español de Oceanografía, Centro Oceanográfico de Murcia, San Pedro del Pinatar, Murcia, Spain; 3 ISPRA Department, for Nature Conservation, Rome, Italy; 4 Department of Ecology, Universidade Federal do Rio Grande do Norte, Natal, Rio Grande do Norte, Brazil; 5 Department of Ecological and Biological Sciences, Ichthyogenic Experimental Marine Center (CISMAR), Tuscia University, Tarquinia, Viterbo, Italy; 6 Accademia del Leviatano, Rome, Italy; 7 Oceanlab, School of Biological Sciences, University of Aberdeen, Newburgh Aberdeenshire, United Kingdom; 8 Departamento de Biologia and CESAM, Universidade de Aveiro, Aveiro, Portugal; 9 Instituto de Investigacións Mariñas (Consejo Superior de Investigaciones Cientificas), Vigo, Spain; 10 Dipartimento di Medicina Veterinaria, Università di Sassari, Sassari, Italy; University of Waikato, NEW ZEALAND

## Abstract

Spatially explicit risk assessment is an essential component of Marine Spatial Planning (MSP), which provides a comprehensive framework for managing multiple uses of the marine environment, minimizing environmental impacts and conflicts among users. In this study, we assessed the risk of the exposure to high intensity vessel traffic areas for the three most abundant cetacean species (*Stenella coeruleoalba*, *Tursiops truncatus* and *Balaenoptera physalus*) in the southern area of the Pelagos Sanctuary, which is the only pelagic Marine Protected Area (MPA) for marine mammals in the Mediterranean Sea. In particular, we modeled the occurrence of the three cetacean species as a function of habitat variables in June by using hierarchical Bayesian spatial-temporal models. Similarly, we modelled the marine traffic intensity in order to find high risk areas and estimated the potential conflict due to the overlap with the cetacean home ranges. Results identified two main hot-spots of high intensity marine traffic in the area, which partially overlap with the area of presence of the studied species. Our findings emphasize the need for nationally relevant and transboundary planning and management measures for these marine species.

## Introduction

Marine spatial planning (MSP) has the potential to provide a comprehensive framework for managing multiple uses of the marine environment (e.g. marine traffic and fishing) and to minimize environmental impacts while reducing conflicts among users [1]. Spatially explicit risk assessment is an essential component of MSP as it links the distribution of key species to the potential effects and distribution of human activities [2, 3].

Cetaceans are recognized *umbrella species* and protecting them could have strong effects on community structure and function [4]. Indeed, it was recently demonstrated that the management of protected areas designed on top predator distributions is highly efficient, leading to higher biodiversity levels and more ecosystem benefits [5, 6]. Consequently, protecting cetacean habitats should be a priority issue for MSP as their protection could act as indirect measures for the management of seas in general [7].

Nowadays cetacean populations are facing several threats including habitat loss, interactions with commercial fisheries, and physical and acoustic disturbance caused, particularly, by increased boating and shipping traffic [8, 9]. Specifically, marine traffic can cause long-term changes in cetacean distribution [10, 11], as well short-term changes in respiration patterns, surface active behaviors and swimming velocity [12, 13]. In addition, worldwide ship strikes with odontocetes and mysticetes are regularly reported, with evidence of ship collisions described for 11 species of large whales, of which the fin whale (*Balaenoptera physalus*) was the most frequently involved [14]

The Mediterranean Sea is a high intensity vessel traffic area in which almost 222,000 vessels, including ferries and fishing boats, navigate daily [15]. The highest intensity of boat traffic occurs in the summer months, especially for the transit of cruise ships and passenger ferries connecting tourist destinations [8, 16]. In addition, the intensity of vessel traffic in European Mediterranean waters is expected to increase over the next few years due to the application of the EU program on “Motorways of the Sea” as an alternative to land transport [17].

In this context it is essential to identify the highest intensity traffic areas that may overlap critical cetacean habitats, in order to provide potential conservation/mitigation measures to protect these species and plan future traffic monitor programs.

In the western Mediterranean sea, most traffic connecting the main ports of central Italy with western destinations passes northern Sardinia through the Bonifacio Strait, a remarkable natural area overlapping several locations with different levels of protection: the Pelagos Sanctuary, the International Marine Park of Bonifacio Bouche, the SPAMI Natural Reserve of *Bouches* de *Bonifacio*, the Asinara and the Maddalena Marine Protected Areas.

In this study, we conducted a spatially explicit assessment of the risk of the exposure to high intensity vessel traffic areas for the three most abundant cetacean species in northern Sardinia [18], striped dolphin (*Stenella coeruleoalba*), bottlenose dolphin (*Tursiops truncatus*) and fin whale (*Balaenoptera physalus*). In particular, we modeled the occurrence of every cetacean species as a function of habitat variables (sea surface temperature, net primary production, photosynthetically active radiation, chlorophyll-a concentration, depth, slope, distance from the coast), by using hierarchical Bayesian spatial-temporal models. Similarly, we modelled the marine traffic intensity in order to find high risk areas, and estimated the overlap with the cetacean area of presence as a measure of potential conflict.

## Material and methods

### Study area

**Fig 1:**
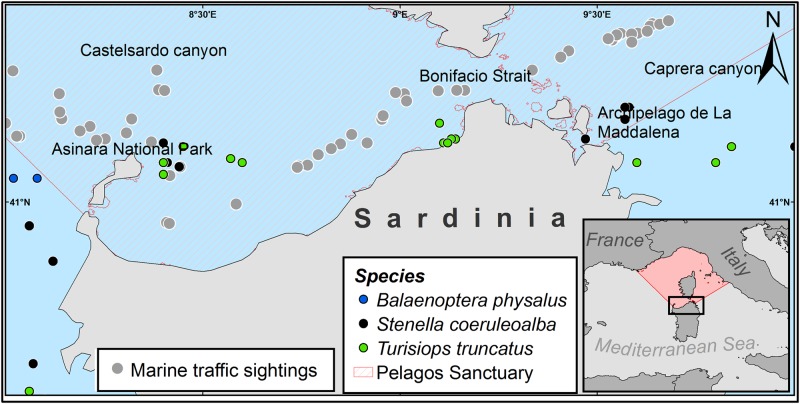
Map of the study area with the boundaries of the Pelagos Sanctuary and the cetaceans and marine traffic observations.

The study area covers approximately 5,500 km^2^ and includes the coastal (0–200 m depth) and offshore (200–3000 m depth) waters of northern Sardinia. Specifically, this area is located between the National Park of the Archipelago de la Maddalena on the eastern side of the island, and the Asinara National Park on the western side (Fig 1).

Part of the studied area is part of the Pelagos Sanctuary, which is the only pelagic MPA for marine mammals in the Mediterranean Sea. It covers about 90,000 km^2^ and was established by Italy, France and Monaco in 1999 [18]. In addition, the study area also encompasses the Natural Reserve of *Bouches* de *Bonifacio* (France), which was listed in 2009 as a specially protected area to protect biological diversity in the Mediterranean (under the SPA/BD Protocol of the Barcelona Convention) [19], and the Bouches de Bonifacio International Marine Park, which, in 2012, adopted measures for the implementation of EU environmental policies at cross-border level [20].

### Cetacean surveys

From 2012 to 2014 a scientific study was performed in June using a motorized-sailing boat (S1 File). During daylight hours (local time from 6.00 A.M. to 8.00 P.M.), quadruple-observer surveys were conducted by trained observers following a boat speed of 8–10 knots. Observers scanned with the naked eye and used binoculars (7 x 50 and 8 x 42) for the identification of cetaceans and estimation of group size. Tracks were recorded using a GPS and daily downloaded onto a computer equipped with the “*Mapsource*” software [21].

Weather conditions (sea state and wind intensity) were recorded every 30 minutes or whenever any of these values changed significantly. Information collected, following the distance sampling protocol [22], included species identification, calf/juvenile presence, group size (maximum, minimum, or exact number if possible), and behaviour of the focal animal identified.

No specific permission was required to perform the sampling. The International Union for Conservation of Nature (IUCN) considers that the striped dolphin and the fin whales are vulnerable and endangered respectively. However, observations were made from a distance of >20 metres in order to avoid harassment of cetaceans [23]. If cetaceans approached the boat, we maintained its course, avoiding abrupt changes in direction or speed to avoid running over or injuring the animals.

### Marine traffic sampling method

Real-time data on marine traffic (S1 File) were collected from 2013 to 2014 within the international Fixed Line Transect network (FLT MED) [24]. Dedicated observers, located on the command bridge of ferries crossing the study area longitudinally, performed scan sampling to count all vessels longer than 5 metres all around the ferry at regular intervals of (every 60 minutes) [8]. Observations were performed only in good weather conditions (Beaufort scale ≤3). In these cases the scan sampling has a range of almost 18 km (calculated with onboard instruments) [25]. The data collected included the occurrence of marine traffic, the geographical position and the type of the boats (boats > = 20 m, boats < = 5m, sailing boats and fishing boats).

Data collection for species and vessel traffic were performed separately, allowing good data sets to be collected on both cetaceans and boats.

### Environmental variables

In order to model the cetacean distribution we used satellite environmental variables that were considered as potential and/or already known predictors of the studied species [26, 27]. These included Sea Surface Temperature (SST in °C), Photosynthetically Active Radiation (PAR in μn), Chlorophyll-a concentration (CHL in mg/m^-3^) and Net Primary Production (NPP in mg C/m^-3^/day), depth (in metres), slope (in degrees) and distance to land (in metres).

Bathymetric features (depth, slope and distance to land) were also used as possible predictors of the marine traffic distribution. We hypnotize that depth and the slope could reflect the morphology of the seabed that could affect the vessels’ distribution. Similarly, distance to land could be correlated to the port proximity and consequently could influence the marine traffic distribution.

The SST, PAR and CHL data were derived from the aqua-MODIS sensor, as daily values with a spatial resolution of 2 km (http://oceandata.sci.gsfc.nasa.gov/), while NPP was obtained using the Windows Image Manager Software [28] with the same spatial resolution.

Depth, slope and distance to land were retrieved from the MARSPEC database, (http://www.marspec.org), with a spatial resolution of 1 km.

All environmental data were gridded at 4 x 4 km in order to have the same spatial resolution using the ‘*raster*’ package [29] in the R software [30].

Finally, environmental variables were explored for linearity, correlation, collinearity, outliers, and missing data before their use in the analysis [31]. In addition, variables were standardized in order to reduce correlation among model coefficients and to enable comparison of relative weights between them. Variables were not highly correlated (r < 0.6), and did not show collinearity or no-linearity, thus they have all been considered in further analyses.

### Statistical models

#### Cetacean distribution

Using the grid index features tool in ArcGIS (version 10.3) [32] a grid of 4 x 4 km cells was created for the studied area. The amount of survey effort in a grid cell, wind intensity and sea state were considered to be the main variables acting on detection probability. Search effort was quantified as the number of times that a cell was visited. For the wind intensity and sea state values, a weighted average was calculated for all the grid cells.

The probabilities of occurrence of the three cetacean species (*S*. *coeruleoalba*, *T*. *truncatus*, *B*. *physalus*) were modeled separately using Bayesian site-occupancy intrinsic conditional autoregressive (*i*CAR) models in order to take into account both imperfect detection and spatial autocorrelation [33]. Two integrated processes were modeled using this type of model: (1) an ecological process associated with the occurrence of the species due to habitat features, and (2) an observation process that takes into account the fact that the probability of detection of the species is less than one [34].

The model of the ecological process includes the environmental variables and an *i*CAR model to account to the spatial autocorrelation between observations [35]. Specifically, if a random variable z_i_ follows a Bernoulli distribution, it can take the value 1 or 0 depending on the habitat suitability i.e. z_i_ = 1 or z_i_ = 0, then:

$$Z_{i}\;\sim \;Bernoulli\left(\pi_{i}\right)$$

$$logit\left(\pi_{i}\right)=X_{i}\beta +\rho_{j\left(i\right)}$$

where *X*_*i*_ is the matrix of covariates, *β* represents the vector of the regression coefficients, ρ represents the spatial random effect of observation *i* in grid cell *j* (i.e. matrix of neighbors), and a logit link is used to model the relationship between the probability of occurrence π_i_, the covariates of interest and spatial effect.

The model of the observation process includes three explanatory variables to account for the variability of the observability of the cetacean species. These variables are the search effort, wind intensity and sea state (Beaufort scale). In particular, the random variable *y*_*it*_ represents the presence of the cetacean species at site *i* and time *t*. The species is observed at site *i* (∑_*t*_ *y*_*it*_ ≥ 1) only if the habitat is suitable (z_i_ = 1). The species is not observed at site *i* (∑_*t*_ *y*_*it*_ = 0) if the habitat is not suitable (z_i_ = 0), or if the habitat is suitable (z_i_ = 1), but the probability *δ*_*it*_ of detecting the species at site *i* and time t is less than 1. Thus, *y*_*it*_ is assumed to follow a *Bernoulli* distribution of parameter z_i_δ_*it*_:

$$y_{it}\;\sim \;Bernoulli\left(z_{i}\delta_{it}\right)$$

$$logit\left(\delta_{it}\right)=W_{it}Ɣ$$

where W_*it*_ includes the explanatory variables and *Ɣ* is the intensity of the process.

Uninformative priors centered at zero with a fixed large variance of 100 were used for all parameters involved in both ecological and observation processes, while a uniform distribution was used for the variance of the spatial effects.

Models were fitted using the “*hSDM*.*siteocc*.*iCAR”* function of the “hSDM” package [36] in R statistical software.

Model selection was performed using all the possible combination of variables and interaction terms. We chose the model which had the lowest Deviance Information Criterion (DIC) [37]. Lower values of DIC represent the best compromise between fit and estimated number of parameters.

Model validation was performed through an internal 10-fold cross validation based on randomly selected training and test datasets (created by a random selection of 75% and 25% of the data respectively), as advised by [38] with the ‘*PresenceAbsence*’ package in R [39]. The area under the receiver-operating characteristic curve (AUC) [40] and the True Skill Statistic (TSS) [41] were used as criteria to assess the goodness of fit of the predictions.

#### Marine traffic distribution

Presence/absence data for marine traffic were gridded (4 x 4 km grid cells) and modeled using an intrinsic conditional autoregressive (*i*CAR) model. Data were aggregated all together and not for type of boats as the total number of observations was not sufficient to achieve a good convergence of the models. We assumed that the response variable z_i_ is a binary variable that can represent the presence (1) or absence (0) of a vessel in each surveyed location, then:

$$Z_{i}\;\sim \;Bernoulli\left(\pi_{i}\right)$$

$$logit\left(\pi_{i}\right)=X_{i}\beta +\rho_{j\left(i\right)}$$

where *X*_*i*_ is the matrix of covariates, *β* represents the vector of the regression coefficients, ρ represents the spatial random effect of the observation *i* at the grid cell *j*, and the logit link is used to model the relationship between π_i_, the covariates of interest and spatial effect. Bathymetry, slope and distance to land were used as possible predictors of the marine traffic distribution.

Models were fitted using the “*hSDM*.*binomial*.iCAR” function of the “hSDM” package within the R statistical software. We followed the same procedure of model selection and model validation as previously for the cetacean models.

All maps, both for cetaceans and marine traffic were generated using the ArcGIS (version 10.3) [32] and the Natural Earth database [42].

#### Testing the overlap between species distribution and marine traffic hot-spots

Predictions of the cetacean distribution and of the marine traffic were compared using the similarity statistics Shoener’s D and Warren’s I [43]. These statistics range from 0 (no overlap between areas), to 1 (distributions are identical). Both statistics assume probability distributions defined over geographic space, in which p_Xi_ (or p_Yi_) denotes the predicted probability assigned by the models for a specific species X and the marine traffic Y to cell *i*. Specifically, Shoener’s statistic for niche overlap is:

D(px, py)=1− 12∑i | pXi−pYi |

while Warren’s statistic is:

I(px, py)=1 12H2(px−py)

which is based on the Hellinger distance (H), defined as:

$$H\left(px,\;py\right)=\;\surd \sum_{i}{\left(\sqrt{pX_{i}}-\sqrt{pY_{i}}\right)}^{2}$$

These analyses were carried out using the *nicheOverlap* function of the *dismo* package [43] in R software.

## Results

### Detection conditions and survey effort

Detection conditions were overall very good, with 92% of effort navigated at sea state and wind intensity 2 or lower. The effort of the cetacean surveys covered 4,863 km^2^ in 2012, 4,367 km^2^ in 2013 and 4,478 km^2^ in 2014. A total of 61 sightings was observed over the analyzed period. Striped dolphin (*S*. *coeruleoalba*) was sighted 27 times, the bottlenose dolphin (*T*. *truncatus*) 24 times and fin whales (*B*. *physalus*) 10 times.

For the marine traffic, 58 scan sampling records were registered within the study period for a total of 323 ships detected. No vessels were observed in 15% of records, while in 4% of records only one ship was present in the studied area. The highest values, ranging from 44 up to 58 vessels counted within the same scan, represented only three cases located in the central part of the Bonifacio Strait.

Among the detected vessels, 73.37% were sailing boats, 14.86% were vessels >20 metres in length, 4.64% were fishing boats and the remaining 7.12% were boats < 5 metres.

### Cetacean distributions

Cetacean distributions in northern Sardinian waters were mainly driven by bathymetry and Sea Surface Temperature (SST), followed by Net Primary Production (NPP) and slope. On the contrary, Photosynthetically Active Radiation (PAR), Chlorophyll-a concentration (CHL) and distance to the coast were found to be irrelevant for cetacean distributions in this area (S1 Table). A positive relationship was found between SST and the occurrence of striped dolphin and fin whales (Table 1) although the final model with the best fit for bottlenose dolphins did not include SST (Table 1).

**Table 1:** Summary of the fixed effects posterior distribution for the best model of the three studied species.

Species	Predictor	Mean	SD	Q_0.025_	Q_0.975_
*T*. *truncatus*	Intercept	-7.46	1.92	-8.79	-4.03
	Depth	-2.24	0.89	-1.05	-0.79
	Slope	-1.62	0.79	-2.18	-0.04
	NPP	0.52	0.47	1.18	2.18
*S*. *coeruleoalba*	Intercept	-5.69	1.48	-6.86	-3.37
	Depth	4.45	1.03	2.67	6.87
	Slope	0.54	0.03	0.75	2.84
	SST	2.74	1.12	1.92	5.13
	NPP	1.02	0.75	0.61	2.13
*B*. *physalus*	Intercept	-3.95	0.78	-4.45	-2.53
	Depth	6.55	0.98	5.84	8.56
	Slope	0.75	0.08	0.45	3.54
	SST	3.63	0.92	2.02	4.97
	NPP	1.54	0.45	0.42	2.02

SST = Sea Surface Temperature; NPP = Net Primary Production. This summary contains the mean, the standard deviation (SD), and a 95% credible interval, which is a central interval containing 95% of the probability under the posterior distribution (Q_0.0025_-Q_0.975_).

NPP showed a positive relationship with the occurrence of all three species, and in particular with fin whale and striped dolphin (Table 1). A positive relationship was observed for bathymetry and slope with the occurrence of fin whale and striped dolphin, *i*.*e* higher occurrence in deeper waters and over seabeds with higher slope. On the contrary, the bottlenose dolphins showed a negative relationship with both variables (Table 1).

**Fig 2:**
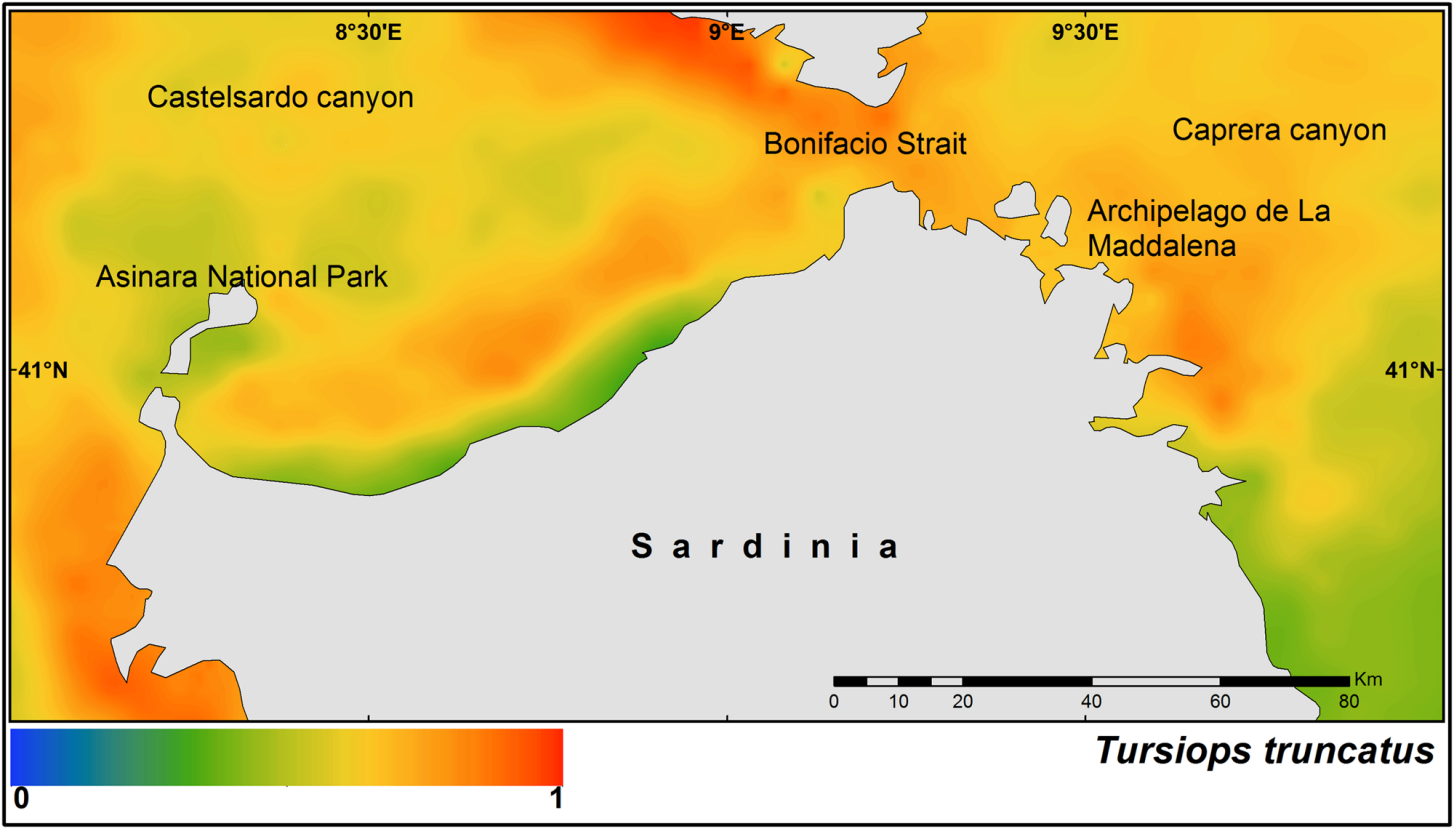
Median of the posterior probability of the presence of the bottlenose dolphin (*Tursiops truncatus*).

The median posterior probability of the occurrence of bottlenose dolphin in northern Sardinian waters is shown in Fig 2.

**Fig 3:**
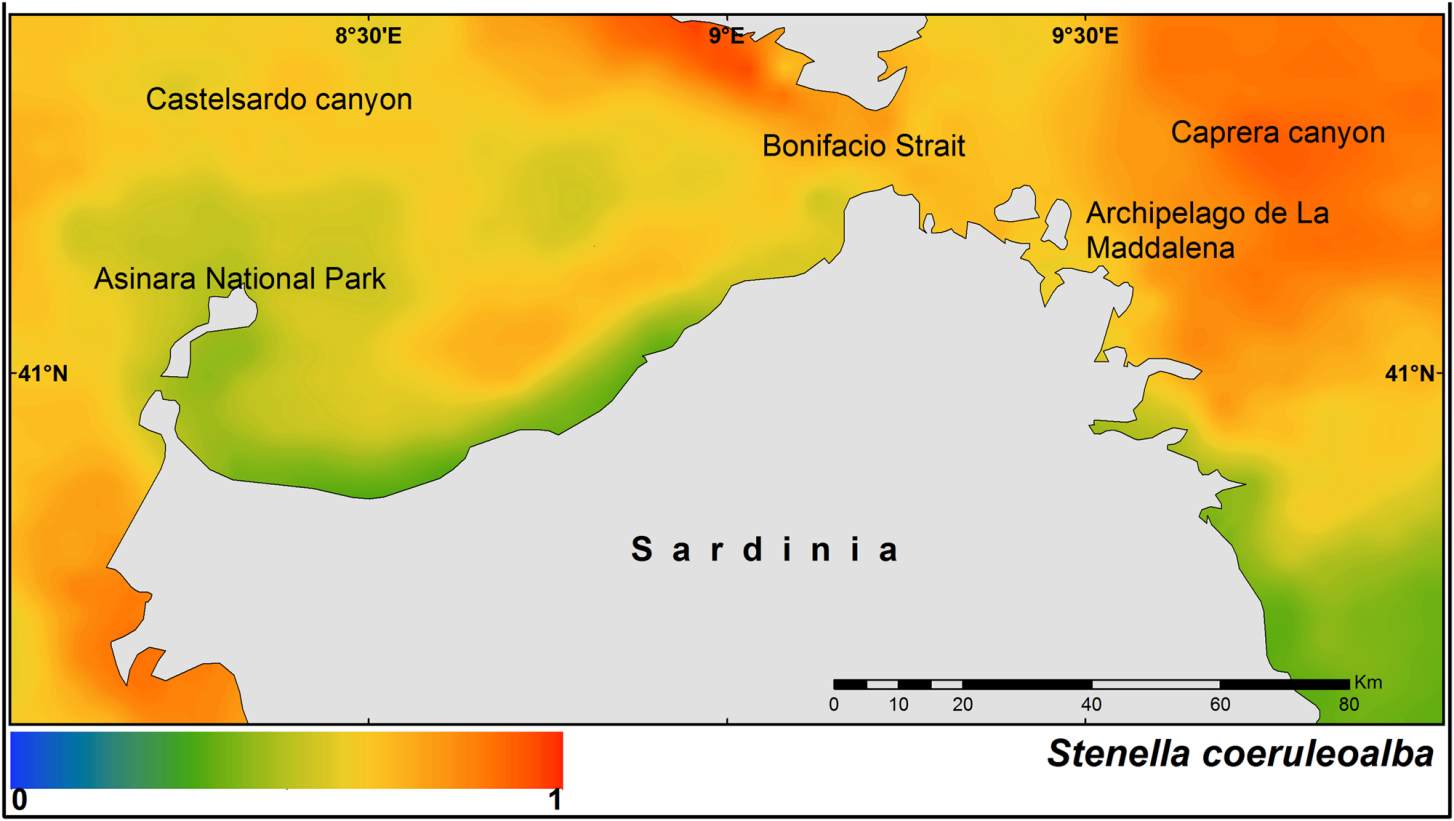
Median of the posterior probability of the presence of the stripped dolphin (*Stenella coeruleoalba*).

Higher probabilities were found on the western side of the study area, in the Bonifacio Strait, and around the Archipelago de La Maddalena. A similar pattern was observed for the striped dolphin, although higher probability of occurrence was predicted on the eastern side of the study area (Fig 3).

**Fig 4:**
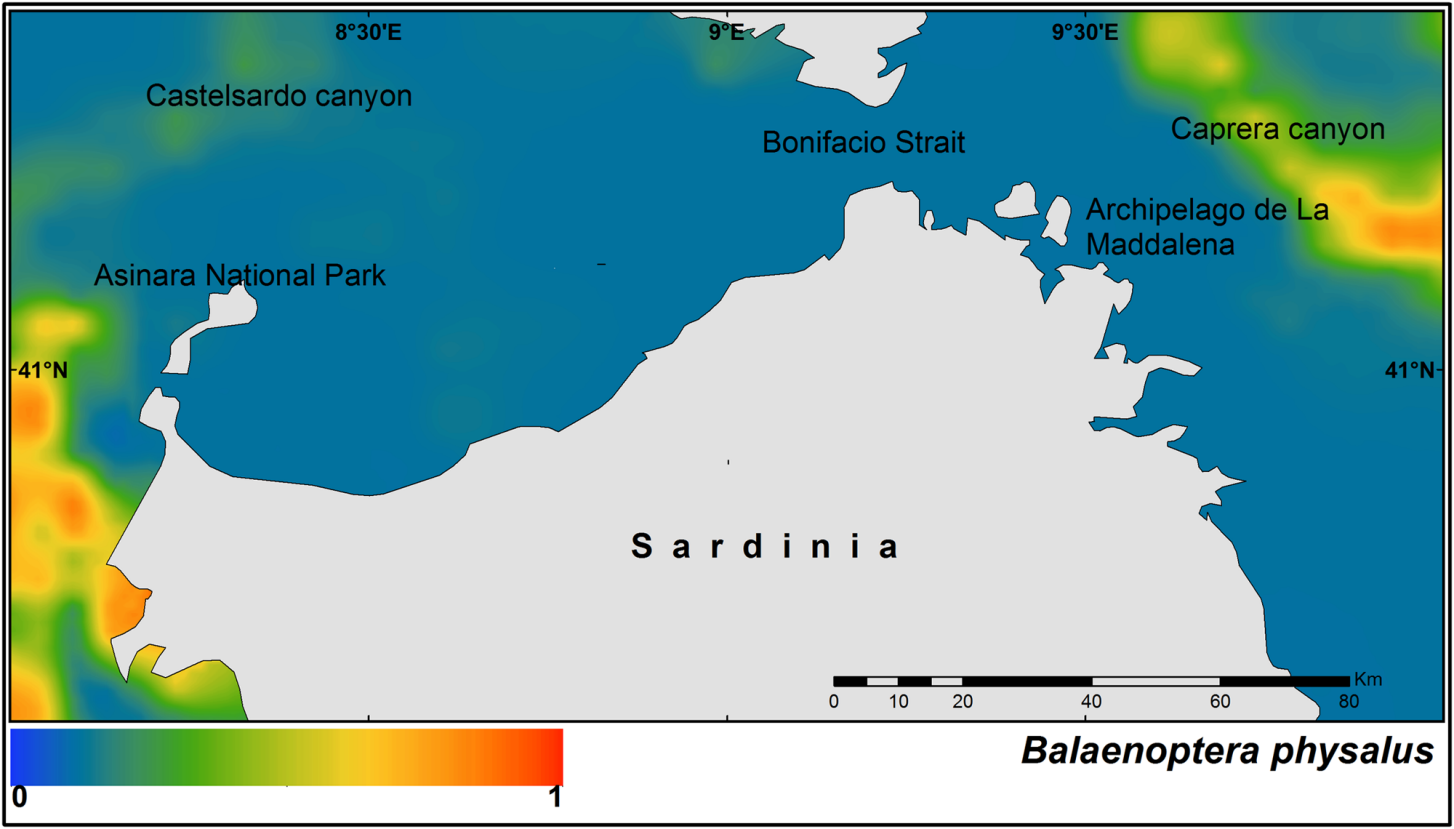
Median of the posterior probability of the presence of the fin whales (*Balaenoptera physalus*).

The fin whale shows two main hot-spots of occurrence, one on the western side of the study area, and another in Caprera Canyon on the eastern side (Fig 4).

For all species, the probability of detection increased with increased effort, and decreased with increasing Beaufort Sea state and wind intensity (Table 2). The detection probability was 0.31 for bottlenose dolphin, 0.35 for striped dolphin and 0.53 for fin whale.

**Table 2:** Estimated coefficients and standard errors (SE) of the variables that influence the detection probability for the observation processes for the three studied species.

Species	Effort	SE	Beaufort sea state	SE	Wind intensity	SE
*T*. *truncatus*	0.18	0.02	-0.26	0.07	-0.45	0.09
*S*. *coeruleoalba*	0.15	0.03	-0.23	0.10	-0.34	0.12
*B*. *physalus*	0.22	0.04	-0.43	0.23	-0.32	0.08

All models showed good values of the predictions measures. Specifically, the bottlenose dolphin was the species that achieved the highest values with an AUC of 0.88 and TSS of 0.83. Striped dolphin and fin whale showed similar results, with an AUC of 0.74 and 0.73, and a TSS of 0.65 and 0.72, respectively.

### Marine traffic

The final model (based on the lowest DIC) of the marine traffic included only the distance to the coast and the spatial effect (S2 Table). Specifically, a positive relationship was found between the occurrence of marine traffic and the distance to the coast (posterior mean = 0.67; 95% IC [0.21, 0.98]).

**Fig 5:**
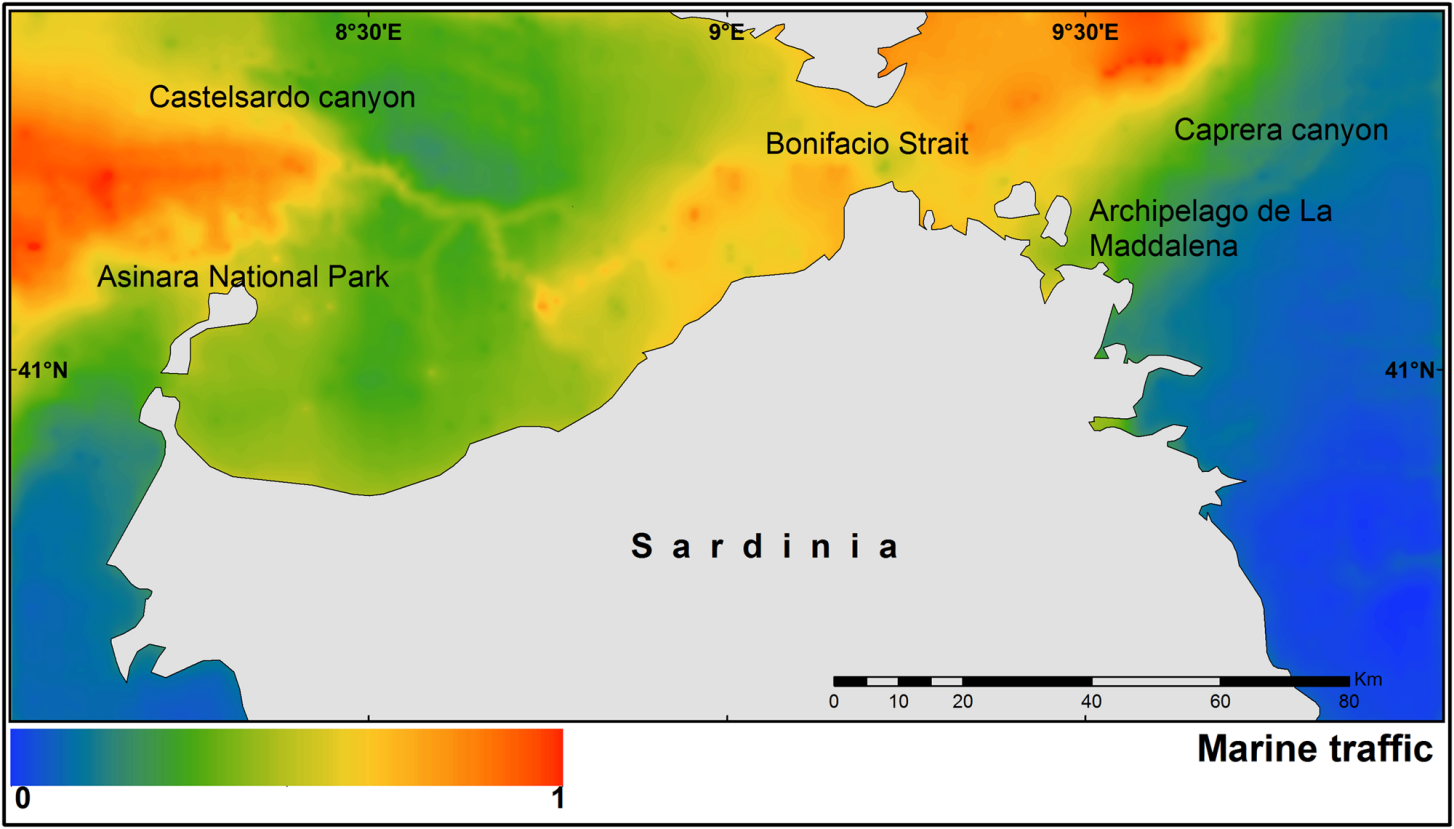
Median of the posterior probability of the marine traffic intensity.

Fig 5 shows the median posterior probability occurrence of the marine traffic in northern Sardinia and highlights two main hot-spots. One is in the Bonifacio Strait and another is in waters surrounding the Asinara National Park.

### Model comparison statistics

Among the three species, the striped dolphin showed the highest overlap with the marine traffic distribution (I = 0.74; D = 0.62). The bottlenose dolphin showed a similar pattern to the striped dolphin with I statistic of 0.73, and a D measure of 0.60. The fin whale had the lowest overlap (I = 0.64; D = 0.55).

## Discussion

Marine Spatial Planning (MSP) offers an opportunity to reduce negative impacts on natural resources and, particularly, on vulnerable species, from marine uses. The first step is the understanding of the spatial extension of species distribution and their hot-spots (e.g., seasonal distribution) and of their possible stressors (e.g., fisheries, marine traffic, etc.). Although some progress has been made in this direction, more studies are needed for marine mammals, to map the overlap of mammal home ranges and stressors spatial distributions to investigate the species. This study provides a first approximation, identifying and synthesizing the available information for the three most abundant cetacean species in northern Sardinia (*S*. *coeruleoalba*, *T*. *truncatus*, *B*. *physalus*) during the June month, as well as the intensity of marine traffic. With regard to the marine traffic models worth to be mentioned that in principle “habitat models” can be constructed for almost anything but the interpretation of the models is clearly likely to be different for living organisms and man-made objects. However, fishing boats may well be found in the same places as cetaceans, because they are following fish. In this sense a similar rationale does apply. In our study, a majority of the boats recorded were recreational boats, and the type of habitat sought by yachtsmen may also be amenable to modelling, although this is less relevant for ferries. Ideally, therefore this approach should be used separately for different classes of boat and further research on this might be useful.

Results identified two main hot-spots of high intensity marine traffic in the area, which partially overlap with the distribution of the studied species. Specifically, the fin whale was the species that showed the lowest degree of spatial overlap with marine traffic, although there was substantial overlap for all three species. This partially overlapping pattern is similar with the one found by [8, 44] in the western Mediterranean Sea. The fin whale is particularly vulnerable to shipping collision due to its biological characteristics and size [14, 15]. This could be due to the fact, as suggested by [14] and [15] that during the June month, whales engage in intensive feeding activities, and may be focused on their prey and less aware of approaching boats. Thus the lower overlap than is seen in the two dolphin species could imply that its habitat selection makes it less likely to encounter boat traffic than is the case for the dolphins.

The distribution of the fin whale also confirms the strong relationship of this species with highly productive areas, probably for feeding purposes. Indeed one of the two main hot-spots of its distribution was identified over the Caprera submarine canyon.

Striped dolphin is the species with the highest degree of overlap between its summer distribution and the high intensity marine traffic areas. This species was also the most frequently observed species within our study area and is known to be regularly sighted in the Mediterranean Sea during June, approaching vessels [45, 46]. Again, habitat choice may account for higher overlap with vessels, and actively approaching vessels will tend to increase the associated collision risk. However, results of [8] in offshore areas of the Western Mediterranean basin reported striped dolphin sightings in patches with lower traffic intensity, likely indicating a different response at a wider scale.

In the same way as for striped dolphin, the bottlenose dolphin showed a high degree of spatial overlap with the high intensity marine traffic areas, especially in the area of the Bonifacio Strait. However, while the habitats of striped dolphin extend over the high seas well beyond the study area, bottlenose dolphin habitat is mainly confined to the coastal areas including Bonifacio Strait. This area is thus particularly critical as it is also frequently used by ships crossing between the western and eastern Mediterranean Sea.

Although there is overlap in the suitable habitat of the three species, each of them presents a different spatial pattern. In addition, the high heterogeneity in the species’ habitat use poses challenges for planning measures aimed at reducing the overlap between cetacean presence and marine traffic. Indeed, selection of a shipping route that has the lowest risk for one species could increase the risk for other species. A careful weighing of priorities is needed when planning the spatial use of the maritime realm, to adapt to specific local situations. In the study area, for example, priority could be given to developing mitigation measures for bottlenose dolphin as the typical habitat of this species almost entirely overlaps with the main traffic areas. Even if several studies have shown coexistence with shipping and bottlenose dolphins, as seen in Aberdeen harbour [47], and a low risk of collision as this species is fast moving, nevertheless, as showed by a previous study in the area [13], disturbance from marine traffic could generate other types of general problems for this species, such as changes in their behaviour and abundance, and displacement from the area of interaction.

The majority of the marine traffic recorded (73.3%) in this area comprised of sailing boats, while vessels over 20 metres in length represented a further15%. Consequently the most probable impact on cetacean species could be represented by short-term responses such as changes in respiration patterns, surface active behaviours, swimming velocity, inter-individual spacing, approach and avoidance, and displacement from the area of interaction [13]. However, it is worth noting that recent studies that analyze Automatic Identification System (AIS) data [48], highlighted important shipping routes off northern eastern Sardinia Island, inside the area used for cetacean distribution modelling, and thus potentially overlapping cetacean habitats.

For these reasons, alternative management options could be also include the reduction of ship speed when crossing through the identified hot-spot areas, education of skippers, as well as the use of observers on-board. Indeed, Vaes et al. [48] showed that high levels of density are observed during the summer months due to the increased of passenger ferries heading to Corsica and Sardinia.

Our findings emphasize the need for nationally relevant and transboundary planning and management measures for these marine species. Our assessment of potential overlap with marine traffic activities should be extended to cover the whole year, when species are at different stages of the reproductive and feeding cycle, for a better understanding of the dynamics of the species in the study area. Indeed, the data used here were sampled over a limited period of time and in a limited area. Thus the models fitted can only reflect a snapshot view of the species distributions and marine traffic. Further studies are also needed on cetacean responses to different vessel categories. Finally, in order to achieve a complete view of the marine traffic stressor, our approach could be complementary to the analysis of the AIS data as they are mandatory only for ships with gross tonnage of 300 or more, while our database includes ships smaller than 300 gross tonnage.

However, our study has helped to visualize the interactions between cetaceans and vessels during the analyzed period, which is the most sensitive time due to the higher intensity of marine traffic. Our results could serve to focus a greater effort in data collection in the identified critical areas, to enhance knowledge on the potential impacts, and drive the development of effective conservation/mitigation measures.

## Supporting information

S1 Table Models comparison of some of the more relevant models for three species studied. Deviance Information Criterion (DIC) scores measure goodness-of-fit. Lower values of DIC represent the best compromise between fit and estimated number of parameters. The spatial effect is included in all the listed models. The model highlighted in bold is the selected one.
(DOC)

S2 Table Model comparison of the marine traffic models. Deviance Information Criterion (DIC) scores measure goodness-of-fit. Lower values of DIC represent the best compromise between fit and estimated number of parameters. W represents the spatial effect.
(DOCX)

S1 File Database. Cetacean and marine traffic datasets.
(PDF)
