# Supplementary material for: A spatially explicit risk assessment approach: Cetaceans and marine traffic in the Pelagos Sanctuary (Mediterranean Sea)
Source: PLoS One. 2017 Jun 23;12(6):e0179686. doi: 10.1371/journal.pone.0179686 (PMC5482452; doi:10.1371/journal.pone.0179686)
Supplement: S1 Table — Deviance Information Criterion (DIC) scores measure goodness-of-fit. Lower values of DIC represent the best compromise between fit and estimated number of parameters. The spatial effect is included in all the listed models. The model highlighted in bold is the selected one. (DOC) [file pone.0179686.s001.doc]

| Species | Model | DIC |
| --- | --- | --- |
| *T. truncatus* | **π(Depth+Slope+NPP), *δ*(effort+SS+WI)** | 362 |
|  | π(Depth+Slope), *δ*(effort+SS+WI) | 375 |
|  | π(Depth+Slope+Chl), *δ*(effort+SS+WI) | 375 |
|  | π(Depth+Slope+PAR), *δ*(effort+SS+WI) | 377 |
|  | π(Depth+Distance+NPP), *δ*(effort+SS+WI) | 382 |
|  | π(Distance+Slope+NPP), *δ*(effort+SS+WI) | 385 |
|  | π(Depth+Slope+SST), *δ*(effort+SS+WI) | 388 |
|  | π(Distance+Slope+NPP+SST), *δ*(effort+SS+WI) | 389 |
|  | π(Depth+Distance+Slope+PAR+Chl+SST+NPP), *δ*(effort+SS+WI) | 424 |
|  | π(Depth), *δ*(effort+SS+WI) | 434 |
|  | π(Slope), *δ*(effort+SS+WI) | 436 |
|  | π(Distance), *δ*(effort+SS+WI) | 439 |
|  | π(PAR), *δ*(effort+SS+WI) | 440 |
|  | π(Chl, *δ*(effort+SS+WI) | 440 |
|  | π(SST), *δ*(effort+SS+WI) | 441 |
|  | π(NPP), *δ*(effort+SS+WI) | 438 |
| *S. coeruleoalba* | **π(Depth+Slope+SST+NPP), *δ*(effort+SS+WI)** | 358 |
|  | π(Depth+Slope+NPP), *δ*(effort+SS+WI) | 365 |
|  | π(Depth+Slope+SST), *δ*(effort+SS+WI) | 367 |
|  | π(Depth+SST+NPP), *δ*(effort+SS+WI) | 370 |
|  | π(Slope+SST+NPP), *δ*(effort+SS+WI) | 382 |
|  | π(Depth+Slope+SST+PAR), *δ*(effort+SS+WI) | 386 |
|  | π(Depth+Slope+SST+Chl), *δ*(effort+SS+WI) | 395 |
|  | π(Depth+Distance+SST+NPP), *δ*(effort+SS+WI) | 398 |
|  | π(Distance+Slope+SST+NPP), *δ*(effort+SS+WI) | 401 |
|  | π(Depth+Slope+SST+NPP+Chl+PAR+Distance), *δ*(effort+SS+WI) | 405 |
|  | π(Depth), *δ*(effort+SS+WI) | 410 |
|  | π(Slope), *δ*(effort+SS+WI) | 415 |
|  | π(Distance), *δ*(effort+SS+WI) | 417 |
|  | π(PAR), *δ*(effort+SS+WI) | 418 |
|  | π(Chl, *δ*(effort+SS+WI) | 418 |
|  | π(SST), *δ*(effort+SS+WI) | 413 |
|  | π(NPP), *δ*(effort+SS+WI) | 414 |
| *B. physalus* | **π(Depth+Slope+SST+NPP), *δ*(effort+SS+WI)** | 298 |
|  | π(Depth+Slope+NPP), *δ*(effort+SS+WI) | 307 |
|  | π(Depth+Slope+SST), *δ*(effort+SS+WI) | 309 |
|  | π(Depth+SST+NPP), *δ*(effort+SS+WI) | 312 |
|  | π(Slope+SST+NPP), *δ*(effort+SS+WI) | 321 |
|  | π(Depth+Slope+SST+PAR), *δ*(effort+SS+WI) | 325 |
|  | π(Depth+Slope+SST+Chl), *δ*(effort+SS+WI) | 328 |
|  | π(Depth+Distance+SST+NPPl), *δ*(effort+SS+WI) | 332 |
|  | π(Distance+Slope+SST+NPP), *δ*(effort+SS+WI) | 338 |
|  | π(Depth+Slope+SST+NPP+Chl+PAR+Distance), *δ*(effort+SS+WI) | 389 |
|  | π(Depth), *δ*(effort+SS+WI) | 392 |
|  | π(Slope), *δ*(effort+SS+WI) | 397 |
|  | π(Distance), *δ*(effort+SS+WI) | 397 |
|  | π(PAR), *δ*(effort+SS+WI) | 399 |
|  | π(Chl, *δ*(effort+SS+WI) | 341 |
|  | π(SST), *δ*(effort+SS+WI) | 394 |
|  | π(NPP), *δ*(effort+SS+WI) | 394 |
