# Supplementary material for: A spatially explicit risk assessment approach: Cetaceans and marine traffic in the Pelagos Sanctuary (Mediterranean Sea)
Source: PLoS One. 2017 Jun 23;12(6):e0179686. doi: 10.1371/journal.pone.0179686 (PMC5482452; doi:10.1371/journal.pone.0179686)
Supplement: S2 Table — Deviance Information Criterion (DIC) scores measure goodness-of-fit. Lower values of DIC represent the best compromise between fit and estimated number of parameters. W represents the spatial effect. (DOCX) [file pone.0179686.s002.docx]

| Model | DIC |
| --- | --- |
| π(Depth + Slope + Distance to the coast + W) | 456 |
| π(Depth + Slope + Distance to the coast) | 523 |
| π(Depth + Slope + W) | 428 |
| π(Depth + Distance to the coast+ W) | 435 |
| π(Slope + Distance to the coast + W) | 436 |
| π(Slope + W) | 402 |
| π(Depth + W) | 398 |
| **π(Distance to the coast + W)** | **387** |
| π(W) | 421 |
