# Supplementary material for: A spatially explicit risk assessment approach: Cetaceans and marine traffic in the Pelagos Sanctuary (Mediterranean Sea)
Source: PLoS One. 2017 Jun 23;12(6):e0179686. doi: 10.1371/journal.pone.0179686 (PMC5482452; doi:10.1371/journal.pone.0179686)
Supplement: S1 File — Cetacean and marine traffic datasets. (PDF) [file pone.0179686.s003.pdf]

1. **Marine traffic dataset**

Boat < 5m	Sailing boats	Fishing vessels	Vessel >20m	Latitude	Longitude
0	0	0	1	41,3325	8,019
1	1	0	0	41,0912	8,7431
0	0	2	1	41,42	9,5474
0	6	0	1	41,3829	9,4273
0	0	0	1	41,5895	10,0977
1	0	0	0	41,0672	8,4166
0	0	0	1	41,1671	8,0363
0	0	0	0	41,1915	8,2144
2	1	0	1	40,9482	8,4087
0	0	0	0	41,1472	8,8492
4	2	0	4	41,2829	9,1638
0	1	0	3	41,4274	9,5593
0	1	2	1	41,0889	8,4425
0	2	0	0	41,2096	8,9541
0	0	0	0	41,452	9,6395
5	1	0	0	41,15	8,9167
0	0	0	1	41,5667	10,05
0	4	0	1	41,1714	8,8778
0	0	0	0	41,4455	9,6334
0	0	0	0	41,2111	8,143
4	0	0	0	40,9458	8,4154
0	3	0	0	41,0935	8,7653
0	0	0	1	41,418	9,5439
0	0	0	1	41,4107	9,5376
0	0	0	1	41,2107333	8,31988333
0	13	0	0	41,0876667	8,73953333
0	0	0	2	41,54225	9,96025
0	16	1	0	41,2183667	8,95701667
0	0	0	2	41,42805	9,6029
0	0	0	0	41,3344167	8,3835
0	10	1	0	41,4258833	9,58663333
0	0	0	1	41,2843333	8,38825
0	0	1	1	41,1666667	8,02865
0	0	0	0	41,2745667	8,13666667
2	2	2	1	41,367	9,40718333
0	0	0	1	41,5416667	9,93128333
0	0	0	0	41,2278667	8,1377
0	0	0	1	41,405	9,58765
0	6	0	0	41,2830833	9,14351667
0	1	0	1	41,1748167	8,3064
0	2	0	1	40,9946833	8,58483333
0	1	0	2	41,4327167	9,62695

0	0	0	2	41,1802833	8,18886667
0	7	0	0	41,3342167	9,3349
2	4	0	2	41,1351167	8,82536667
0	2	1	2	41,4508333	9,65828333
0	41	1	2	41,2745833	9,01056667
0	0	0	0	41,1524167	8,37013333
0	8	0	1	41,2826667	8,40305
0	56	2	0	41,2775167	8,98865
0	2	0	0	41,1596167	8,25301667
1	12	0	0	41,2560167	9,01663333
0	26	0	3	41,2818167	9,0968
0	0	0	2	41,45945	9,68041667
0	0	1	0	41,15685	8,23081667
1	5	1	0	41,1203167	8,79303333
0	1	0	1	41,4234667	9,54896667
0	0	0	1	41,4143	9,52756667

2. Cetaceans' dataset

**Latitude**	**Longitude**	**Species**
41.16	9.47	*S. coeruleoalba*
41.21	9.57	*S. coeruleoalba*
41.24	9.58	*S. coeruleoalba*
41.24	9.57	*S. coeruleoalba*
41.24	10.03	*S. coeruleoalba*
41.20	10.03	*S. coeruleoalba*
41.18	10.05	*S. coeruleoalba*
41.18	10.03	*S. coeruleoalba*
41.17	10.04	*S. coeruleoalba*
41.14	10.00	*S. coeruleoalba*
41.14	10.02	*S. coeruleoalba*
41.14	10.03	*S. coeruleoalba*
41.18	10.06	*S. coeruleoalba*
41.19	10.06	*S. coeruleoalba*
41.18	10.05	*S. coeruleoalba*
41.23	10.06	*S. coeruleoalba*
41.15	8.40	*S. coeruleoalba*
41.10	8.41	*S. coeruleoalba*
41.09	8.44	*S. coeruleoalba*
41.07	8.40	*S. coeruleoalba*
40.59	8.07	*S. coeruleoalba*
40.23	8.00	*S. coeruleoalba*
40.23	7.57	*S. coeruleoalba*
41.01	10.06	*S. coeruleoalba*
41.05	10.05	*S. coeruleoalba*
41.07	10.04	*S. coeruleoalba*
41.07	10.08	*S. coeruleoalba*
40.85	8.12	*S. coeruleoalba*
40.94	8.06	*S. coeruleoalba*
41.24	10.03	*B. physalus*
41.19	10.05	*B. physalus*
41.23	10.06	*B. physalus*
40.35	7.6	*B. physalus*
41.06	8.02	*B. physalus*
41.06	8.02	*B. physalus*
41.06	8.08	*B. physalus*
41.22	10.05	*B. physalus*
40.31	8.01	*B. physalus*
41.17	10.1	*B. physalus*
41.20	9.10	*T.truncatus*
41.20	9.10	*T.truncatus*
41.20	9.10	*T.truncatus*

40.50	8.10	*T.truncatus*
40.30	8.10	*T.truncatus*
40.10	8.30	*T.truncatus*
40.20	8.30	*T.truncatus*
41.10	8.60	*T.truncatus*
41.10	8.40	*T.truncatus*
41.10	8.40	*T.truncatus*
40.30	8.10	*T.truncatus*
40.50	8.10	*T.truncatus*
41.10	9.80	*T.truncatus*
41.20	9.10	*T.truncatus*
41.10	9.60	*T.truncatus*
40.90	9.60	*T.truncatus*
41.16	9.14	*T.truncatus*
41.16	9.13	*T.truncatus*
41.15	9.11	*T.truncatus*
40.48	8.10	*T.truncatus*
40.32	8.10	*T.truncatus*
40.13	8.27	*T.truncatus*
40.17	8.28	*T.truncatus*
41.11	8.57	*T.truncatus*
41.14	8.45	*T.truncatus*
41.07	8.40	*T.truncatus*
40.33	8.10	*T.truncatus*
40.52	8.06	*T.truncatus*
41.14	9.84	*T.truncatus*
41.15	9.12	*T.truncatus*
